# Understanding adaptation to fluconazole: comparative insights into tolerance and resistance in *Saccharomyces cerevisiae* and *Candida albicans*

**DOI:** 10.3389/fcimb.2025.1519323

**Published:** 2025-05-05

**Authors:** Lijun Zheng, Yubo Dong, Jing Wang, Yonghui Jia, Weifang Wang, Yi Xu, Liangsheng Guo

**Affiliations:** ^1^ Department of Ultrasound Medicine, The Second Affiliated Hospital of Soochow University, Suzhou, China; ^2^ Department of Pharmacy, The 960th Hospital of PLA, Jinan, China; ^3^ Department of Pharmacy, Zibo Zhoucun People’s Hospital, Zibo, China; ^4^ Jinzhou Medical University Graduate Training Base, The 960th Hospital of PLA, Jinan, China; ^5^ Department of Obstetrics and Gynecology, The Second Affiliated Hospital of Soochow University, Suzhou, China

**Keywords:** fluconazole tolerance, petite, efflux, ergosterol, *Saccharomyces cerevisiae*

## Abstract

**Introduction:**

Antifungal resistance and tolerance are distinct responses exhibited by fungi when exposed to drugs. While considerable research has focused on azole tolerance in the human pathogen *Candida albicans*, studies in other fungal species remain limited.

**Objective:**

This study aims to conduct a comparative investigation of the adaptation of the model organism *Saccharomyces cerevisiae* and *C. albicans* to fluconazole *in vitro*.

**Methods:**

We performed experiments using laboratory strains of *S. cerevisiae* and *C. albicans* to evaluate their fluconazole tolerance and resistance under varying temperature conditions. High concentrations of fluconazole were administered, and subsequent changes in fungal phenotypes were analyzed through techniques such as transcriptome analysis and monitoring of petite formation.

**Results:**

Our results revealed that fluconazole tolerance is present in wild-type strains of *S. cerevisiae* and is influenced by temperature, albeit in a manner opposite to that observed in *C. albicans*. Importantly, when subjected to high concentrations of fluconazole, *S. cerevisiae* strains developed resistance without displaying tolerance; all resistant adaptors identified were petites. Chemical induction of petite formation led to an increase in resistance accompanied by a decrease in tolerance.

**Conclusion:**

Transcriptome analysis indicated that petites up-regulated efflux mechanisms while down-regulating most *ERG* genes. This suggests that, unlike petite-negative *C. albicans*, petite-positive *S. cerevisiae* swiftly transitions to a petite phenotype upon exposure to fluconazole, resulting in enhanced resistance but diminished tolerance. This evolutionary divergence emphasizes the need for additional studies on fluconazole tolerance in other pathogenic fungi.

## Introduction

Fungal infections represent a significant global health burden, affecting over one billion individuals worldwide (Bongomin et al., 2017). Immunocompromised and critically ill patients are particularly vulnerable, with fungi ranking among the leading causes of morbidity and mortality in these populations (Pfaller and Diekema, 2007). The World Health Organization (WHO) recently underscored the urgency of this issue by listing 19 fungal pathogens as “priority pathogens,” including *Candida albicans*, *Cryptococcus neoformans*, *Aspergillus fumigatus*, and *Candida auris*, which are classified as “critical priority” due to their high public health risk (WHO, 2022). While *C. albicans* remains the most common cause of candidiasis, the prevalence of non-albicans Candida (NAC) species in hospital-acquired infections has risen significantly in recent decades, with some species even surpassing *C. albicans* in isolation frequency (Cleveland et al., 2015; Wu et al., 2017). This shift is partly attributed to the widespread use of azole antifungals, which has exerted selective pressure on fungal populations (Mean et al., 2008).

The current antifungal arsenal is limited to four main classes: azoles, polyenes, echinocandins, and pyrimidine analogues (Robbins et al., 2016). Among these, azoles—particularly fluconazole (FLC)—are the most widely used due to their efficacy and relatively low toxicity to host cells (Pfaller et al., 2010). Azoles inhibit lanosterol 14α-demethylase, a key enzyme in the ergosterol biosynthesis pathway, leading to the accumulation of toxic sterols and depletion of ergosterol in fungal cell membranes (Joseph-Horne and Hollomon, 1997; Kanafani and Perfect, 2008). However, because azoles are fungistatic rather than fungicidal, they do not eradicate fungal cells, creating conditions conducive to the emergence of resistance. Since their introduction in the 1980s, the incidence of azole-resistant fungal pathogens has risen alarmingly worldwide (Fisher et al., 2018). For instance, resistance rates to FLC in C. neoformans clinical isolates have increased from 7.3% (1997-2000) to 11.7% (2005-2007) (Pfaller et al., 2009). Similarly, the annual resistance rate of Candida tropicalis to FLC continues to rise, posing a significant challenge to antifungal therapy (Wang et al., 2021).

In addition to resistance, fungi exhibit a phenomenon known as tolerance, which further complicates treatment outcomes. While resistance is defined as the ability to grow at drug concentrations that inhibit susceptible strains, tolerance refers to the ability of susceptible strains to survive prolonged drug exposure without significant growth inhibition (Balaban et al., 2019; Berman and Krysan, 2020). In bacteria, tolerance is quantified by the minimum duration of killing (MDK), but no standardized methods exist for measuring antifungal tolerance in fungi. This lack of consensus has led to inconsistent terminology and methodologies in the literature, hindering progress in understanding fungal responses to treatment (Yang and Berman, 2024).

Recent work by the Berman lab has redefined antifungal tolerance as the ability of drug-susceptible fungal strains to grow slowly at drug concentrations above the minimal inhibitory concentration (MIC) (Rosenberg et al., 2018). Using disk diffusion and broth microdilution assays, they developed metrics such as the radius of inhibition (RAD) and the fraction of growth (FoG) to quantify tolerance (Gerstein et al., 2016; Rosenberg et al., 2018). These studies have revealed that wild-type *C. albicans* isolates exhibit intrinsic azole tolerance, which varies depending on strain characteristics and environmental factors such as temperature, medium composition, and pH (Rosenberg et al., 2018; Delarze et al., 2020; Xu et al., 2021; Yang et al., 2023). Notably, *C. albicans* adapts to high azole concentrations by developing tolerance rather than resistance, whereas low concentrations of FLC promote resistance, sometimes accompanied by tolerance (Sun et al., 2023; Todd et al., 2023; Yang et al., 2023; Zheng et al., 2024b). Furthermore, genes such as *KSR1*, *ERG251*, and *ZRG2* have been implicated in FLC tolerance in *C. albicans* (Vande Zande et al., 2024; Zhou et al., 2024), highlighting the genetic basis of this adaptive response.

Antifungal tolerance has also been observed in other fungal species, including *C. auris* and *C. glabrata* (Rasouli Koohi et al., 2023; Zheng et al., 2024a). However, it remains unclear whether tolerance is a widespread adaptive strategy among fungi or limited to specific species. This knowledge gap highlights the need for further research to elucidate the mechanisms underlying fungal tolerance and its implications for antifungal therapy.

*Saccharomyces cerevisiae*, a well-established model organism in microbiology and genetics, has been instrumental in advancing our understanding of eukaryotic biology and antifungal resistance mechanisms (Demuyser and Van Dijck, 2019). Its genetic tractability and well-characterized pathways make it an ideal system for studying the molecular basis of antifungal responses. However, despite its utility, the adaptive strategies of *S. cerevisiae* in response to azole exposure, particularly the interplay between resistance and tolerance, remain poorly understood.

This study aims to address this gap by investigating the adaptive responses of *S. cerevisiae* to FLC and comparing them to those of *C. albicans*. By elucidating the mechanisms underlying FLC tolerance and resistance in *S. cerevisiae*, we seek to contribute to a broader understanding of fungal adaptation strategies and inform the development of more effective antifungal therapies.

In this study, we compared FLC adaptation in *S. cerevisiae* (S288C) and *C. albicans* (SC5314). While SC5314 exhibited FLC tolerance at 37°C but not 30°C, S288C showed the opposite pattern, being tolerant at 30°C but not 37°C. *S. cerevisiae* adapted to high FLC concentrations primarily through resistance, with all resistant adaptors being petites. Ethidium bromide-induced petites also lost tolerance and gained resistance, accompanied by up-regulation of efflux genes and down-regulation of ERG genes. These findings suggest mitochondrial integrity is crucial for FLC tolerance in *S. cerevisiae*.

## Materials and methods

### Strains and growth conditions

The reference strain *S. cerevisiae* S288C, the clinical isolate MK288, and the *C. albicans* reference strain SC5314 were utilized as progenitors in this study. Stock cultures were preserved in 25% glycerol and maintained at -80˚C. Cells were routinely cultured in Yeast Extract-Peptone-Dextrose (YPD) medium, which consists of 1% (w/v) yeast extract, 2% (w/v) peptone, and 2% (w/v) D-glucose, at designated temperatures using a shaking incubator set to 150-200 rpm. For YPG medium, the composition included 1% (w/v) yeast extract, 0.2% (w/v) peptone, and 3% (w/v) glycerol, with 2% (w/v) agar added for solid media. Drug solutions were prepared in dimethyl sulfoxide (DMSO) and stored at -20˚C.

### Disk diffusion assay

Disk diffusion assays were conducted following the protocols outlined by Yang et al (Yang et al., 2023), adhering to the CLSI M44-A2 guidelines for antifungal disk diffusion susceptibility testing with minor modifications. In brief, strains were streaked from glycerol stocks onto YPD agar and incubated at designated temperatures for 48 hours. Colonies were then suspended in distilled water and adjusted to a concentration of 1 × 10^6^ cells/mL. A volume of 100 μL of this cell suspension was spread evenly onto -m20L YPD plates. An empty paper disk (6 mm diameter and 0.7 mm thickness) was saturated with 5 μL of 40 mg/mL FLC and placed at the center of each plate. The plates were subsequently incubated at the specified temperatures and photographed after 48 hours. Analysis of the disk diffusion assay was performed using the *diskImageR* pipeline (Gerstein et al., 2016). The parameters measured included the fraction of growth within the zone of inhibition (FoG) and the radius of inhibition (RAD), specifically at 20% drug inhibition, denoted as FoG_20_ and RAD_20_, respectively.

### Isolating colonies from zone of inhibition

Cells were suspended in distilled water and adjusted to a concentration of 1 × 10^5^ cells/mL. A volume of 100 µL of the cell suspension was spread onto a YPD plate. An empty paper disk saturated with 5 μL of 40 mg/mL FLC was placed at the center of the plate. After incubating 48 hours at 30˚C for S288C and 37˚C for SC5314, 16 colonies were randomly selected from within the inhibition zone and streaked onto new YPD plates. Following an additional 48-hour incubation, one single colony from each adaptor was chosen for further analysis.

### Obtaining adaptors using high concentrations of fluconazole

Cells were suspended in distilled water and adjusted to a concentration of 1 × 10^7^ cells/mL. A volume of 100 µL of the cell suspension was spread onto YPD plates supplemented with FLC. The plates were then incubated at 30˚C for 5 days. Adaptors were randomly selected from the drug-treated plates.

### Spot assay

Cells were suspended in distilled water and adjusted to a concentration of 1 × 10^7^ cells/mL. A volume of 3 µL of the cell suspension was spotted onto YPD or YPG plates. The plates were incubated at 30˚C and photographed after 48 hours.

### Induction of petite formation in yeast using ethidium bromide

The methodology for inducing petite formation with Ethidium bromide (EtBr) was adapted from the protocol described by Fox et al (Fox et al., 1991), with slight modifications. The test strains were thawed from a -80°C freezer and streaked onto a YPD plate, followed by incubation at 30°C for 48 hours to promote colony growth. A single colony was then inoculated into YPD broth supplemented with 25 µg/mL EtBr (filter-sterilized). Once the culture reached saturation, it was transferred to a second culture prepared with the same medium and allowed to grow to saturation again. For strain S288C, one single passage was performed, while for SC5314, a total of 20 passages were conducted. Finally, the saturated cultures were streaked onto YPD plates to isolate individual colonies, which were subsequently streaked onto both YPD and YPG plates to verify respiratory deficiency.

### RNA-seq

RNA sequencing (RNA-seq) was performed as previously described with minor modifications (Yang et al., 2021). Briefly, strains were streaked on YPD plates from the -80˚C freezer and incubated for 48 hours at 30˚C. Several colonies of similar size were randomly selected and suspended in a solution to achieve an optical density at 600 nm (OD600) of 0.1. The cultures were then incubated in a shaking incubator at 30˚C until the OD600 reached 1.0. Cells were collected by centrifugation and flash frozen in liquid nitrogen.

Total RNA extraction and purification, library construction, and sequencing were conducted as described previously (Ke et al., 2024). Three biological replicates were obtained for each strain. Differential gene expression profiling was performed using DESeq2 (Love et al., 2014) with standard parameters. Genes with a False Discovery Rate (FDR)-adjusted p-value < 0.05 and expression fold changes greater than 1.5 or less than -1.5 were considered differentially expressed.

### Measurement of total ergosterol content

Ergosterol was extracted and detected as described in by Madsen et al (Madsen et al., 2011). Briefly, strains were cultured in YPD broth until reaching the log phase (OD600 = 1.0). The cells were then collected, centrifuged at 4000 rpm for 3 minutes, and the resulting pellets were immediately stored at −20°C. The frozen pellets were thawed and resuspended in 2 ml of a 20% w/v sodium hydroxide solution in 50% ethanol. The mixture was transferred to a glass tube and heated in boiling water for 5 minutes with occasional shaking. After cooling, 1 ml of the same sodium hydroxide solution and 2 ml of hexane were added, followed by vortex-mixing for 30–60 seconds. The tubes were centrifuged at 1000 rpm for 5 minutes, and the hexane phase was collected for further analysis. The samples were dried, derivatized by adding 50 µl of BSTFA and 50 µl of pyridine, dried again, and finally dissolved in 75 µl of toluene.

Ergosterol content was quantified using Gas Chromatography-Mass Spectrometry (GC-MS). A 1 µl sample was injected into an Rtx-5 ms column (30 meters, 0.25 mm ID) with helium as the carrier gas. The column temperature was initially held at 240°C for 2 minutes, then increased at a rate of 10°C per minute to 330°C, and maintained at 330°C for 6.5 minutes.

### Measurement of rhodamine 6G efflux

Rohodamine 6G efflux experiment was performed following the protocol described by Kolaczkowski et al (Kolaczkowski et al., 1996). Briefly, approximately 1 × 10^6^ yeast cells from overnight cultures were transferred to YPD medium and allowed to grow for 4 hours. The cells were then pelleted, washed twice with PBS (pH 7.0, without glucose), and resuspended in glucose-free PBS to a density of 10^8^ cells/ml. The cell suspensions were incubated at 30°C with shaking at 200 rpm for 120 minutes under glucose starvation conditions to de-energize the cells. After de-energization, the cells were pelleted, washed, and resuspended in glucose-free PBS to 10^8^ cells/ml. Rhodamine 6G was added to the suspensions at a final concentration of 10 µM, and the cells were incubated for 30 minutes at 30°C. Following incubation, the cells were washed twice and resuspended in glucose-free PBS to 10^8^ cells/ml. To measure rhodamine 6G efflux, 1-mL samples were collected at 10-minute intervals, centrifuged, and the absorption of the supernatant at 527 nm was measured in triplicate using 100-µl aliquots. Energy-dependent efflux was assessed by adding 2% glucose (final concentration) to the cell suspensions, while glucose-free controls were included in all experiments. The efflux of rhodamine 6G was quantified using a rhodamine 6G concentration curve for accurate determination.

### Statistical analysis

All disk diffusion assays represent the average of three technical replicates, with error bars indicating the standard deviation. Statistical analyses were conducted using a two-tailed Student’s t-test in Microsoft Excel. Significance analysis of total ergosterol content was performed using a two-tailed Student’s t-test to evaluate differences between strains. Curves representing rhodamine 6G concentrations were analyzed using the Tukey HSD test to assess statistical significance. A p-value of less than 0.05 was considered statistically significant. ** indicates p < 0.01, *** indicates p < 0.001.

## Results

### Opposing effects of temperature on fluconazole tolerance in reference strains of *S. cerevisiae* and *C. albicans*

To compare *S. cerevisiae* and *C. albicans* regarding the potential impact of temperature on FLC tolerance, two assays were conducted using reference strains.

**Figure 1:**
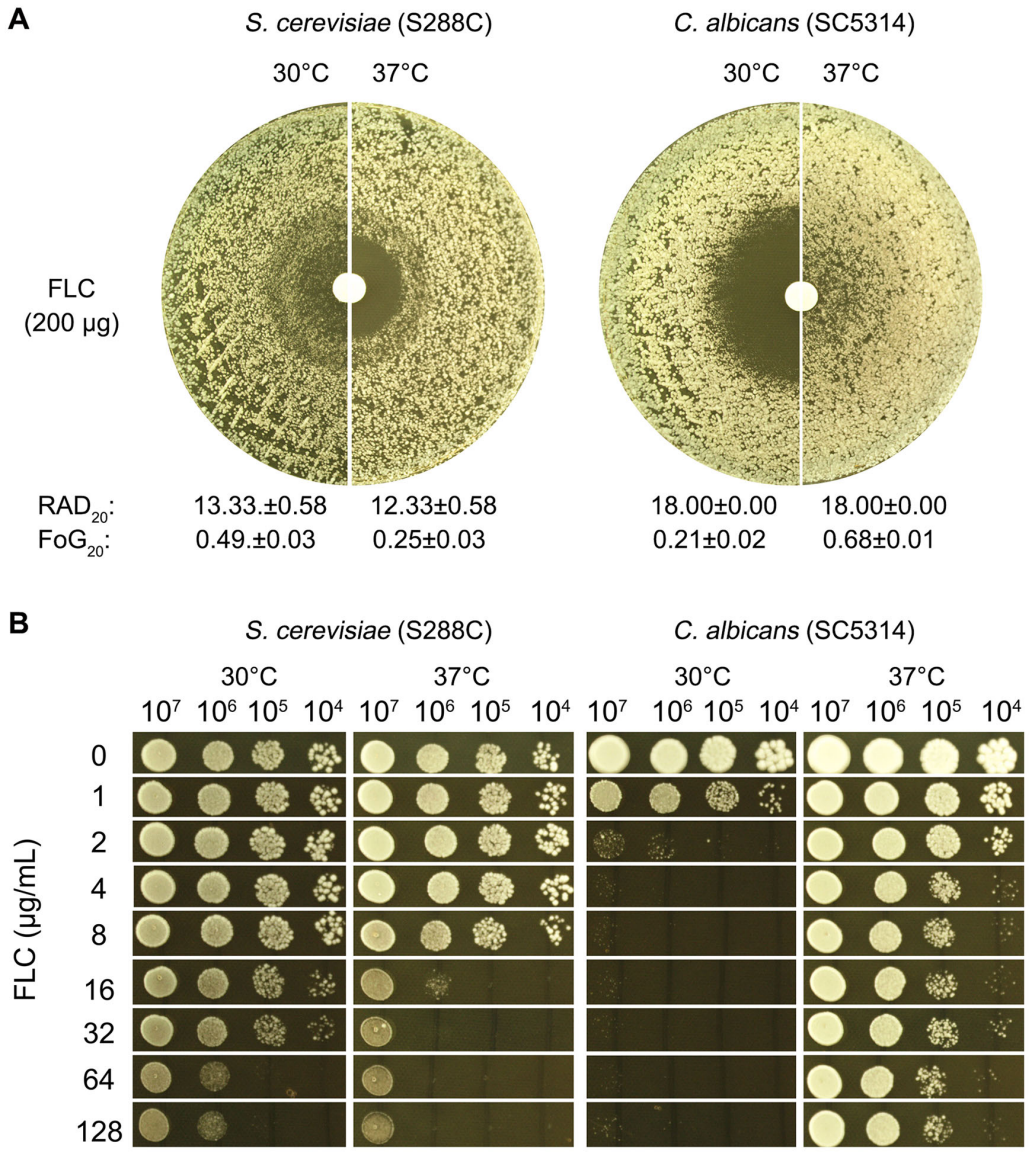
Evaluation of fluconazole tolerance in *S. cerevisiae* and *C. albicans*. To investigate the effect of temperature on FLC tolerance in *C. albicans* and *S. cerevisiae*, two assays were performed. In the disk diffusion assay **(A)**, paper disks containing 200 μg of FLC were placed on YPD-agar plates inoculated with approximately 1 × 10^5^ cells of SC5314 and S288C. The plates were incubated at 30°C and 37°C, as indicated in the figure, for 48 hours. RAD_20_ and FoG_20_ were quantified using the *diskImageR* script. In the spot assay **(B)**, 3 μL of 10-fold serial dilutions were spotted onto YPD-agar plates containing varying concentrations of FLC. These plates were also incubated at 30°C and 37°C for 48 hours before being photographed.

In the disk diffusion assay (**Figure 1A**), S288C had RAD_20_ values of 13.33 ± 0.58 at 30°C and 12.33 ± 0.58 at 37°C. SC5314 exhibited the same RAD_20_ value at both 30°C and 37°C. Thus, temperature did not produce significant changes in RAD_20_ values for either strain. For S288C, a clear ZOI was observed at 37°C with a FoG20 value of 0.25 ± 0.03. In contrast, at 30°C, S288C displayed lawn growth inside the ZOI, yielding a FoG_20_ value of 0.49 ± 0.02, which was significantly higher than the value at 37°C (p<0.001, two-tailed Student’s t-test). SC5314 displayed a clear ZOI at 30°C, with a FoG_20_ value of 0.21 ± 0.02. At 37°C, however, it showed growth within the ZOI, resulting in a higher FoG_20_ value of 0.68 ± 0.01, which was significantly greater than the value at 30°C (p<0.001, two-tailed Student’s t-test).

In the spot assay (**Figure 1B**), serial dilutions of both strains were applied to YPD-agar plates containing varying concentrations of FLC. S288C exhibited better growth at 30°C compared to 37°C in the presence of 16–128 μg/mL FLC. Growth of SC5314 was noticeably inhibited at 2 μg/mL FLC at 30°C, while at 37°C, growth was not substantially affected even at concentrations up to 128 μg/mL FLC.

Overall, these results indicate a significant modulation of FLC tolerance in both *C. albicans* and *S. cerevisiae*, influenced by temperature. This highlights the differential responses of these yeast species to antifungal treatment under varying thermal conditions.

### Impact of calcineurin and hsp90 inhibition on fluconazole tolerance in *S. cerevisiae* and *C. albicans*

Previous studies indicate that calcineurin and heat shock protein 90 (Hsp90) are required for maintaining tolerance to azoles in *C. albicans* (Rosenberg et al., 2018; Xu et al., 2021; Guo et al., 2024; Zheng et al., 2024b) and *C. glabrata* (Zheng et al., 2024a). Calcineurin is a calcium/calmodulin-dependent serine/threonine phosphatase crucial for various cellular processes across different organisms, including bacteria, fungi, and higher eukaryotes. In microorganisms, particularly fungi, calcineurin is essential for regulating responses to environmental stresses such as high salinity, oxidative stress, and antifungal treatments (reviewed in (Yadav and Heitman, 2023)). Hsp90 is a conserved molecular chaperone vital for protein homeostasis, facilitating the folding, stability, and maturation of various client proteins, including kinases and transcription factors. It plays a key role in cellular stress responses under conditions like heat shock and oxidative stress. In fungal pathogens, Hsp90 is critical for antifungal resistance, regulating key proteins involved in drug resistance mechanisms, such as efflux pumps. Inhibiting Hsp90 not only disrupts the function of these proteins but also sensitizes resistant strains to conventional antifungal therapies [reviewed in (Iyer et al., 2022)].

**Figure 2:**
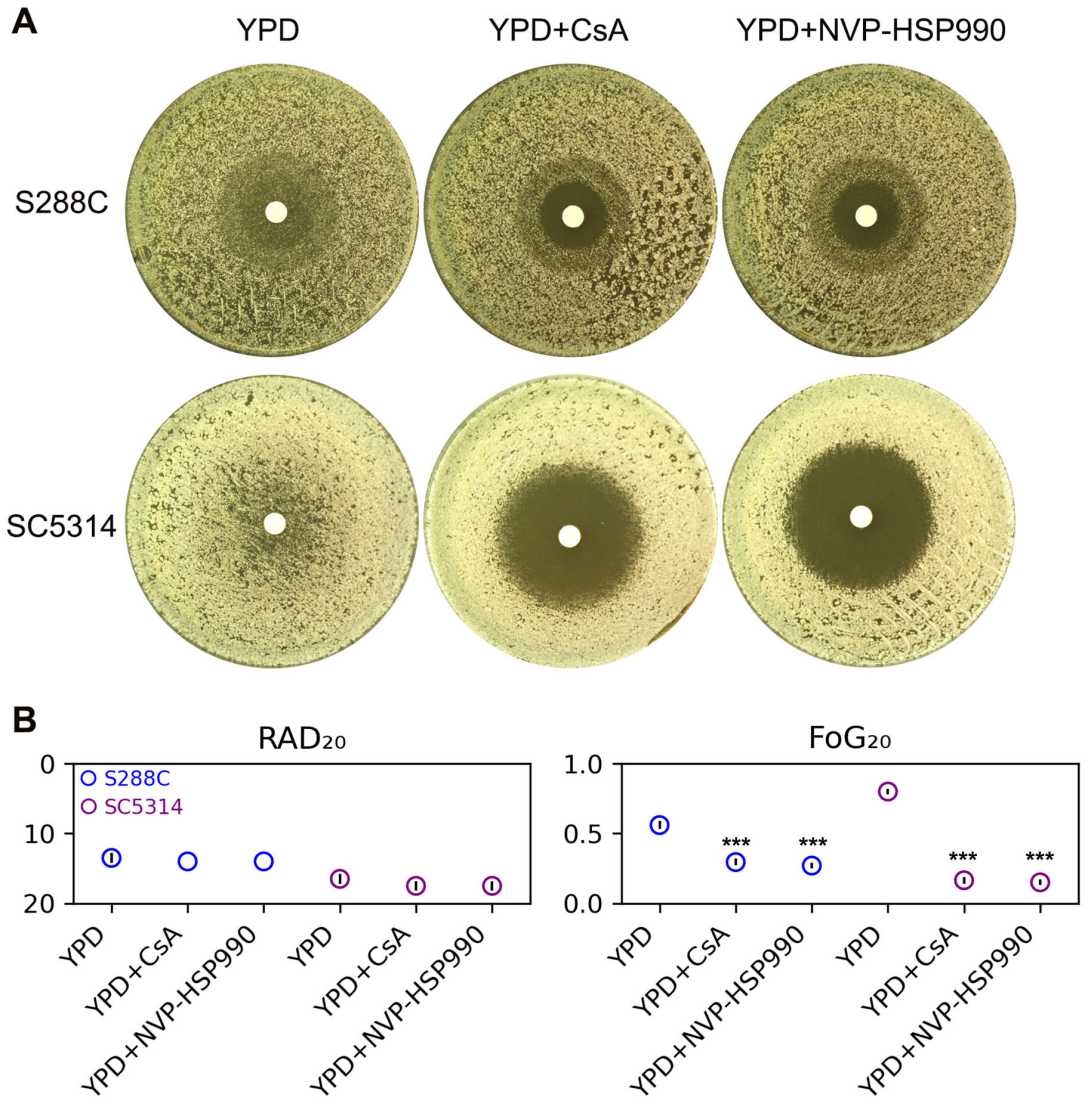
Influence of calcineurin and Hsp90 on fluconazole tolerance. **(A)** Disk diffusion assays were performed on YPD-agar and YPD-agar supplemented with the calcineurin inhibitor cyclosporine A (CsA, 0.5 μg/mL) or the Hsp90 inhibitor NVP-HSP990 (8 μg/mL). Each disk contained 200 μg of FLC. The plates were incubated at 30°C for strain S288C and at 37°C for strain SC5314 for 48 hours before being photographed. **(B)** RAD_20_ and FoG_20_ values were calculated using the *diskImageR* package. These values represent the average of three biological replicates, with error bars indicating standard deviation. Two-tailed Student’s t-test was conducted. *** indicates p < 0.001 in comparison to the values obtained on YPD.

In this study, we investigated whether calcineurin and Hsp90 are required for maintaining FLC tolerance in *S. cerevisiae*. For comparison, we also tested *C. albicans*. In disk diffusion assays, both S288C and SC5314 exhibited lawn growth on YPD media and clear halos on YPD supplemented with the calcineurin inhibitor cyclosporin A or the Hsp90 inhibitor NVP-HSP990 (**Figure 2A**). Quantitative measures indicated that the addition of both inhibitors resulted in a significant decrease in FoG_20_ values (p<0.001, two-tailed Student’s t-test), but no obvious change in RAD_20_ values p>0.05, two-tailed Student’s t-test (**Figure 2B**). Thus, like *C. albicans*, FLC tolerance in *S. cerevisiae* is also dependent on calcineurin and Hsp90.

### Investigating the characteristics of IZO colonies and their resistance/tolerance to fluconazole

The tolerance to FLC is demonstrated by the slow growth of yeast in environments with high concentrations of FLC, resulting in smaller colony sizes compared to those outside the ZOI. Certain yeasts, such as *S. cerevisiae* and *Candida glabrata*, can lose their mitochondria or oxidative respiratory functions under specific conditions. The resulting cells, known as petite mutants, are viable and form small, slow-growing colonies (Fekete et al., 2007). The petite yeasts are unable to grow on nonfermentable carbon sources (such as glycerol) (Day, 2013). Petite *C. glabrata* cells exhibit resistance to azole drugs through the overexpression of ABC transporter genes *CDR1*, *CDR2*, and *SNQ2*, along with their transcriptional regulator *PDR1* (Brun et al., 2004; Tsai et al., 2006). *C. albicans* can also form petite mutants, which show increased resistance to FLC due to the overexpression of the major facilitated superfamily multidrug efflux pump gene *MDR1* (Cheng et al., 2007a).

Therefore, we investigated whether the colonies found within the ZOI (hereafter referred to as IZO colonies) are indeed petites.

**Figure 3:**
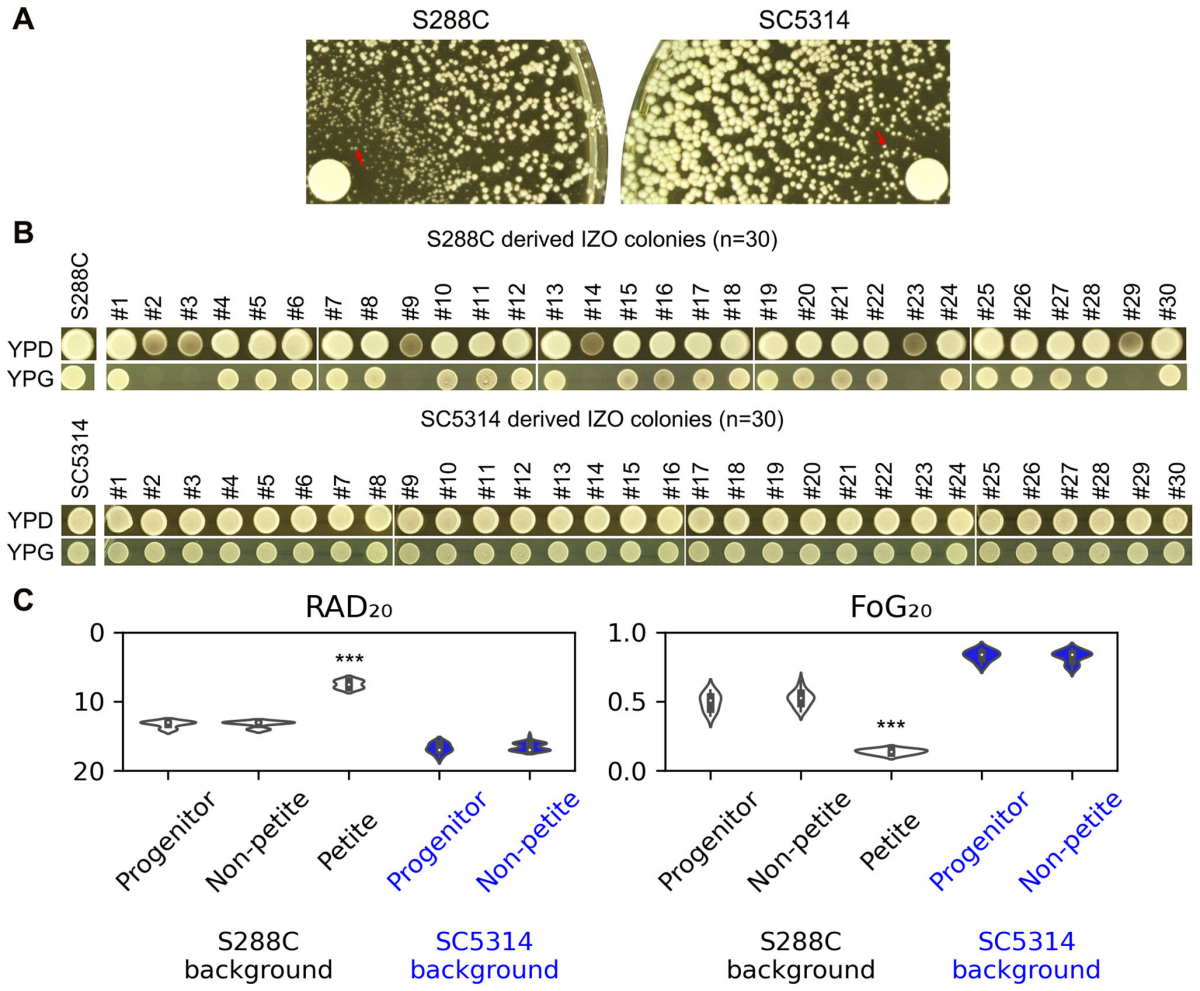
Examination of IZO Colonies. **(A)** Approximately ten thousand cells of S288C and SC5314 were spread on YPD-agar plates. The disks used contained 200 μg of FLC. The plates were incubated at 30°C for S288C and at 37°C for SC5314 for 48 hours to yield IZO colonies. **(B)** Thirty random IZO colonies derived from each progenitor were selected. Both the progenitors and IZO colonies were spotted onto YPD and YPG-agar plates, which contain glucose and glycerol as carbon sources, respectively. **(C)** A disk diffusion assay was performed to evaluate the resistance and tolerance of IZO colonies to FLC. The RAD_20_ and FoG_20_ values presented were generated using the *diskImageR* package. For the progenitors, eight individual colonies were tested, representing eight biological replicates. For the IZO colonies, the data represents the mean of three biological replicates for each colony. *** indicates p < 0.001 compared to the progenitor.

To isolate IZO colonies, a disk diffusion assay was performed using a cell density that was tenfold lower than that typically used in standard assays (**Figure 3A**). After 48 hours, 30 colonies were randomly selected from the zone of inhibition (ZOI). Among the 30 IZO colonies derived from S288C, six were unable to grow on YPG-agar plates, which utilize glycerol as the sole carbon source. In contrast, all 30 IZO colonies derived from SC5314 were able to grow on YPG-agar plates (**Figure 3B**).

The disk diffusion assays indicated that the petites exhibited significantly reduced RAD_20_ and FoG_20_ values compared to S288C (p<0.001, two-tailed Student’s t-test), suggesting that while the petites were more resistant than S288C, they had lost tolerance. The non-petite IZO colonies, derived from either S288C or SC5314, did not show significant changes in RAD_20_ or FoG_20_ values, indicating that they maintained similar levels of resistance and tolerance to FLC when compared to their parental strains (**Figure 3C**).

### Adaptation of *S. cerevisiae* to high concentration of fluconazole

Previous studies have shown that *C. albicans* adapts to high concentrations of azole drugs, including FLC, posaconazole, ketoconazole, and miconazole, primarily through the development of tolerance, as evidenced by increased FoG_20_ values (Kukurudz et al., 2022; Yang et al., 2023; Guo et al., 2024). Recently, we discovered that *C. glabrata* exhibits a parallel evolution of FLC resistance and tolerance, demonstrated by reduced RAD_20_ values and increased FoG_20_ values, respectively (Zheng et al., 2024a). In this study, we investigated how *S. cerevisiae* adapts to high concentrations of FLC. We included the *C. albicans* strain SC5314 as a control in our experiments.

**Figure 4:**
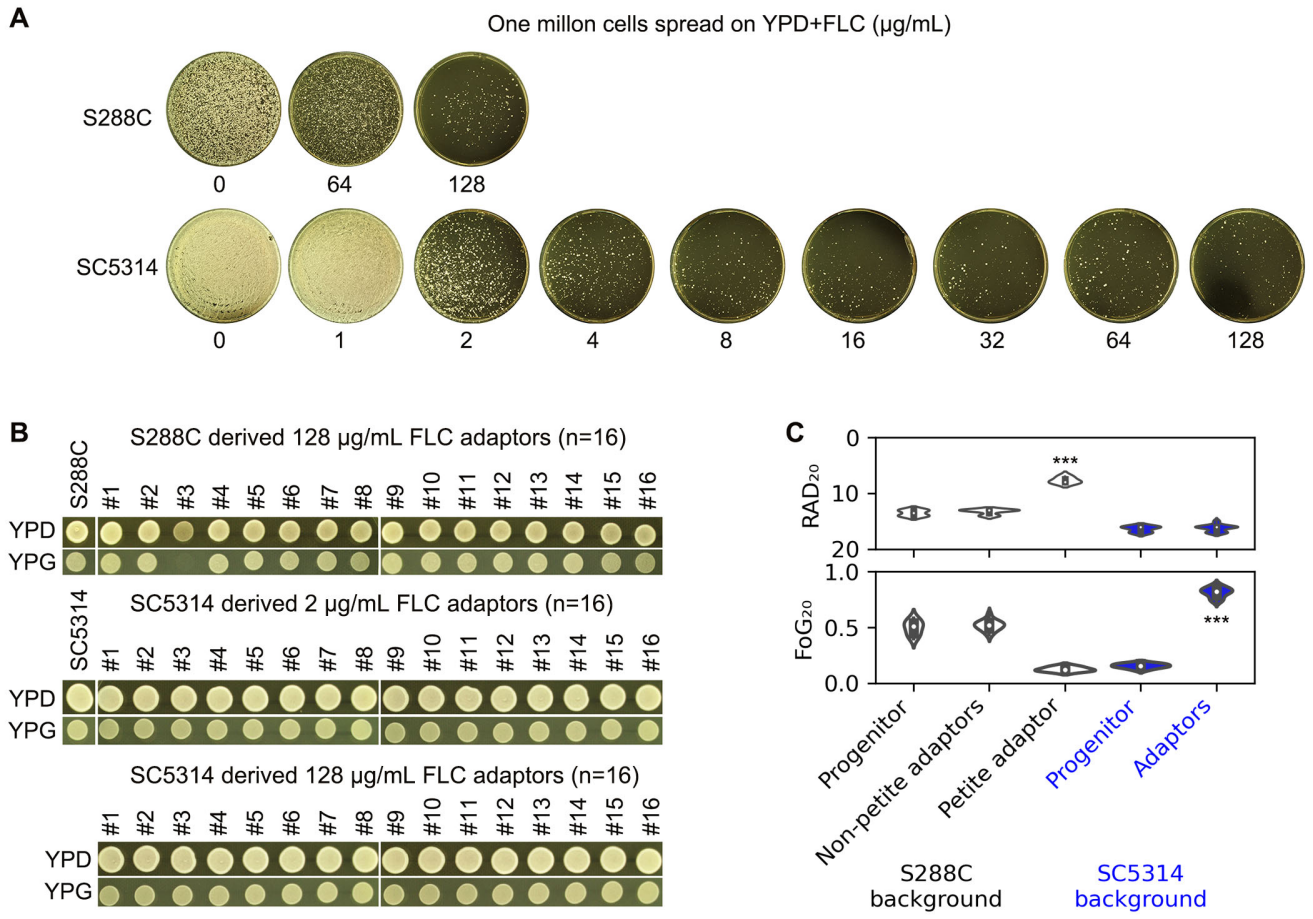
Adaptation of *S. cerevisiae* and *C. albicans* reference strains to fluconazole. **(A)** Approximately one million cells of S288C and SC5314 were spread on YPD-agar plates containing FLC. The drug concentrations are indicated in the figure. The plates were incubated at 37°C for S288C and at 30°C for SC5314 for 5 days. **(B)** Sixteen colonies of *S. cerevisiae* were randomly selected from the 128 μg/mL FLC plate, and 16 colonies of *C. albicans* were chosen from both the 2 and 128 μg/mL FLC plates, respectively. The adaptors were then spotted on YPD and YPG plates, which were photographed after 48 hours of growth. **(C)** A disk diffusion assay was conducted using 200 μg of FLC. For both progenitors and the petite adaptor, eight individual colonies were tested. The mean RAD_20_ and FoG_20_ values from eight biological replicates are presented for the progenitors and petite adaptor, along with the means of three biological replicates for the other adaptors. *** indicates p < 0.001 compared to the progenitor.

S288C and SC5314 were spread on YPD-agar plates containing a wide range of FLC concentrations (8-128 μg/mL for S288C and 1-128 μg/mL for SC5314). At a concentration of 128 μg/mL, FLC exhibited significant inhibitory effects against S288C. FLC concentrations ranging from 2 to 128 μg/mL also demonstrated a strong inhibitory effect against SC5314, resulting in only a few hundred colonies appearing on the plates (**Figure 4A**).

For S288C, we randomly selected 16 colonies (hereafter designated as FLC adaptors) from the plate treated with 128 μg/mL FLC. For SC5314, we randomly picked 16 adaptors from both the 2 μg/mL and 128 μg/mL FLC plates. When tested for growth ability on YPG, one adaptor derived from S288C failed to grow, while all other adaptors did (**Figure 4B**).

The disk diffusion assay indicated that the petite adaptor derived from S288C had significantly reduced RAD_20_ values and decreased FoG_20_ values compared to S288C (p<0.001, two-tailed Student’s t-test), whereas the other adaptors derived from S288C did not exhibit significant changes in RAD_20_ or FoG_20_ values. In contrast, all adaptors derived from SC5314 showed significantly increased FoG_20_ values (p<0.001, two-tailed Student’s t-test) (**Figure 4C**).

**Figure 5:**
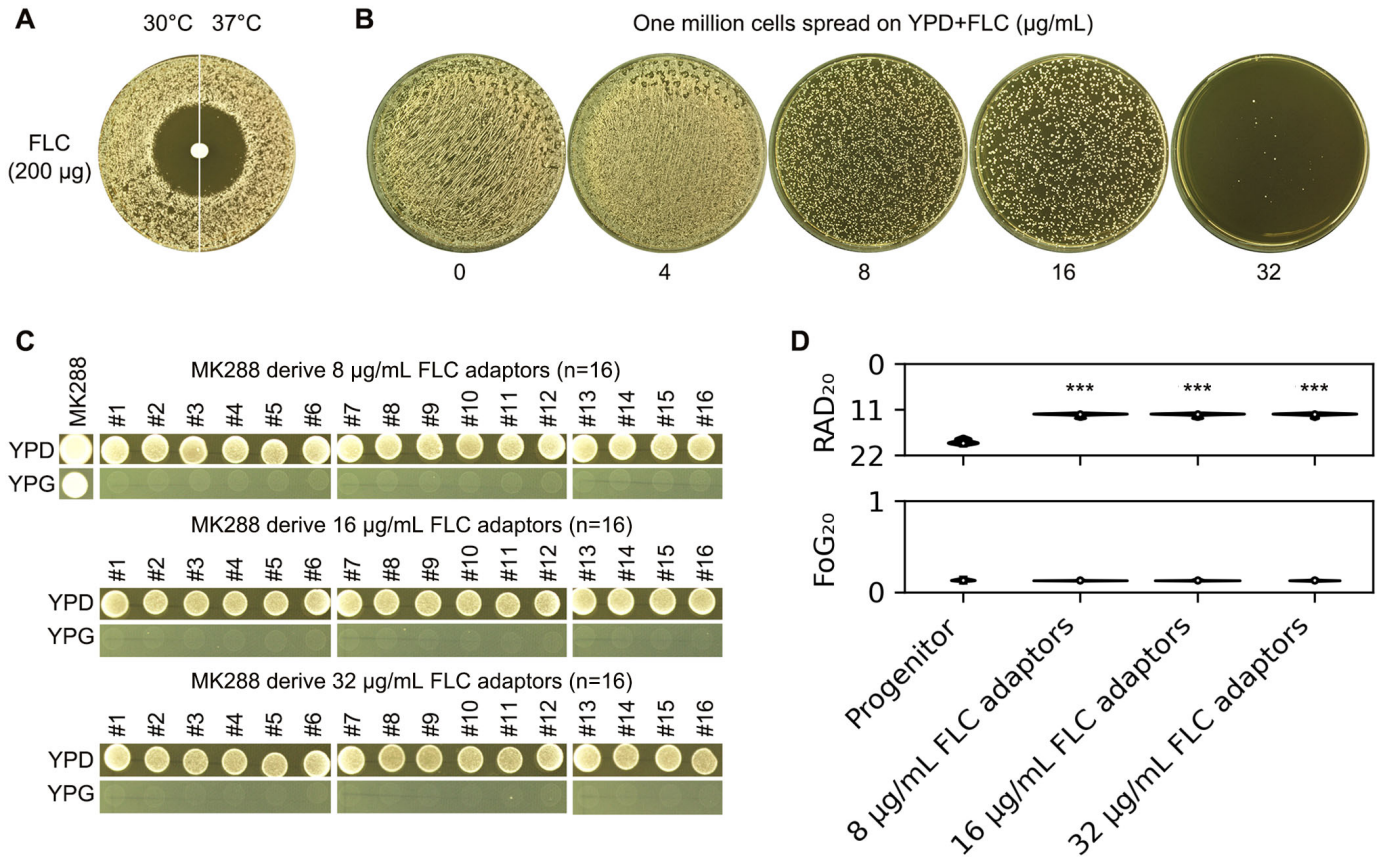
Adaptation of a clinical *S. cerevisiae* isolate to fluconazole. **(A)** The clinical isolate MK288 was evaluated using a disk diffusion assay with disks containing 200 μg of FLC. Two incubation temperatures were tested: 30°C and 37°C. The plates were incubated at the designated temperatures for 48 hours before being photographed. **(B)** Approximately one million cells of MK288 were spread on YPD-agar plates containing 4-32 μg/mL FLC, and the plates were incubated at 30°C for 5 days. Sixteen colonies (adaptors) were randomly selected from each plate containing 8-32 μg/mL FLC. **(C)** The progenitor MK288 and the FLC adaptors were spotted on YPD and YPG-agar plates. Strains were adjusted to 1 × 10^7^ cells/mL, and 3 μL was spotted onto the plates. The plates were incubated at 30°C for 48 hours before being photographed. **(D)** The progenitor and FLC adaptors were also assessed using a disk diffusion assay with disks containing 200 μg of FLC. RAD_20_ and FoG_20_ values were calculated using the *diskImageR* package. For the progenitor, eight individual colonies were tested as biological replicates, while three colonies representing three biological replicates were tested for each adaptor. Mean values are shown. *** indicates p < 0.001 compared to the progenitor.

In addition to S288C, we evaluated how a clinical isolate of *S. cerevisiae* adapted to FLC. Unlike S288C, whose tolerance to FLC was influenced by temperature, the clinical isolate MK288 exhibited no tolerance at either 30°C or 37°C (**Figure 5A**). Approximately one million cells of MK288 were spread on YPD-agar plates containing FLC. From the plates with 8-32 μg/mL FLC, 16 colonies were randomly selected from each concentration (**Figure 5B**). While the progenitor could grow on YPG, none of the adaptors were able to do so, indicating that all adaptors were petite (**Figure 5C**). The disk diffusion assay revealed that all adaptors had significantly reduced RAD_20_ values compared to the progenitor (p<0.001, two-tailed Student’s t-test), while FoG_20_ values showed no significant changes (**Figure 5D**). Thus, MK288 adapted to FLC primarily through petite formation and the development of resistance

In summary, FLC primarily induces resistance in *S. cerevisiae* and tolerance in *C. albicans*. The resistant *S. cerevisiae* adaptors are petite, whereas the tolerant *C. albicans* adaptors are not.

### Loss of fluconazole tolerance in S288C due to ethidium bromide-induced petite formation: a contrast with SC5314

**Figure 6:**
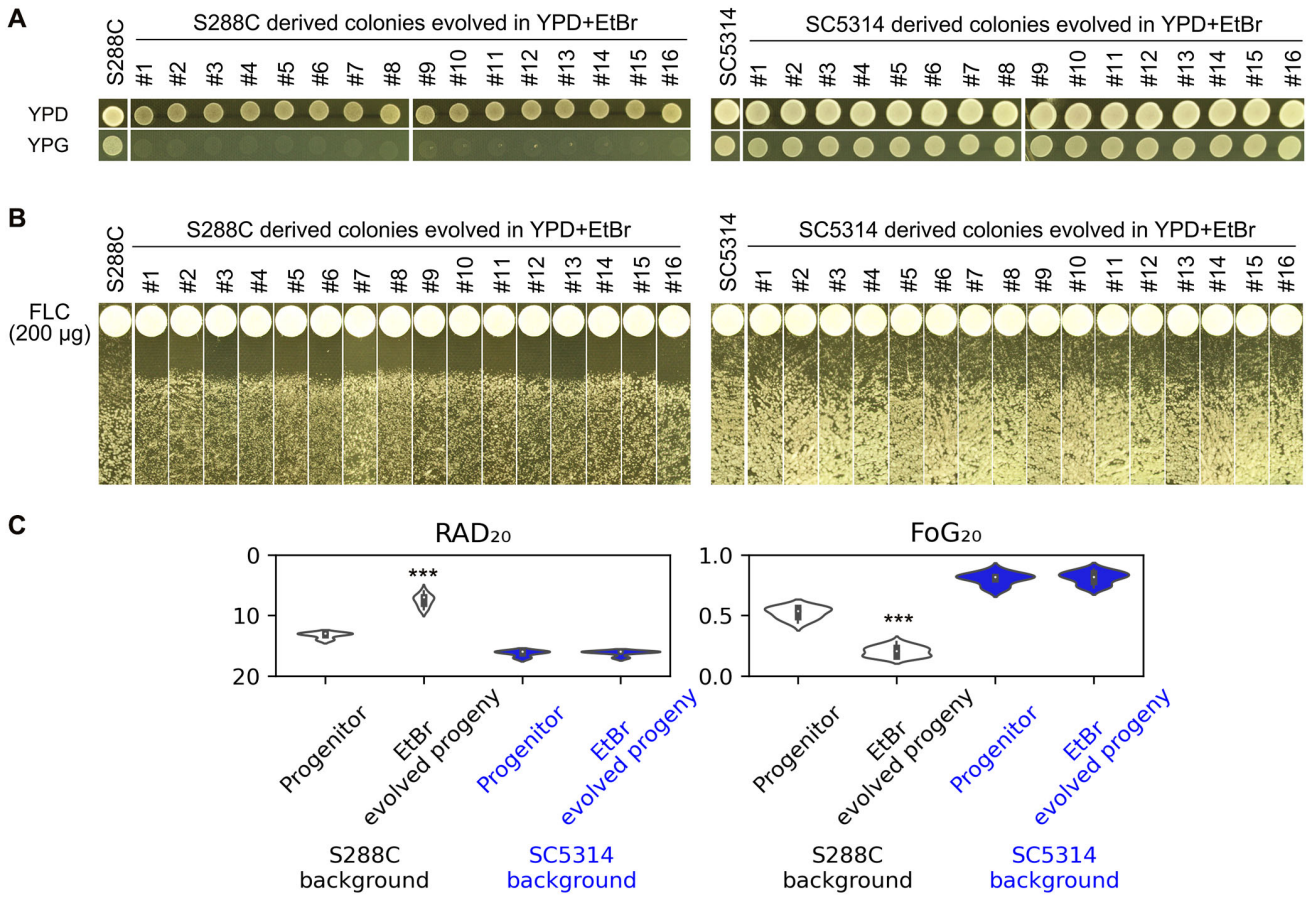
Impact of Ethidium Bromide on Fluconazole Tolerance and Resistance. **(A)** S288C and SC5314 strains were passaged in YPD media supplemented with EtBr. The resulting progeny was then spotted onto YPD and YPG plates to assess respiratory deficiency. **(B)** The progeny evolved from EtBr exposure were subjected to a disk diffusion assay using disks containing 200 μg of FLC to evaluate their sensitivity to the drug. **(C)** Quantification of the disk diffusion assay results was performed using the *diskImageR* package. For parental strains, eight individual colonies were analyzed as biological replicates, while three individual colonies were assessed for each progeny. Statistical significance is indicated by *** (p < 0.001) when compared to the progenitor strains.

From the above experiments, we observed that S288C, which was initially tolerant to FLC, lost its tolerance after becoming petites upon exposure to FLC. To further investigate the requirement for intact mitochondrial respiratory function in relation to FLC tolerance, we employed an alternative method to induce petite formation. Ethidium bromide (EtBr) is known to inhibit mitochondrial DNA synthesis and induce the degradation of pre-existing mitochondrial DNA, thereby converting respiratory-sufficient yeast into respiratory-deficient petites (Goldring et al., 1970). S288C was cultured in YPD medium containing EtBr, and after a single passage, 16 random colonies were selected for analysis. None of these colonies were able to grow on YPG (**Figure 6A**), which is consistent with previous reports indicating that even short-term exposure to EtBr is sufficient to induce petite formation in Saccharomyces cerevisiae (Fox et al., 1991). In contrast, SC5314 was grown in YPD medium supplemented with EtBr for 20 passages, and all 16 randomly chosen colonies retained the ability to grow on YPG. This finding supports the notion that *C. albican*s exhibits a low propensity for petite formation (Chen and Clark-Walker, 2000). Disk diffusion assays demonstrated that all EtBr-evolved petite progeny derived from S288C exhibited clear and reduced ZOI, whereas the EtBr-evolved progeny of SC5314 showed lawn growth within the ZOI (**Figure 6B**). Quantitative measurements indicated that the S288C-derived progeny had significantly decreased levels of FoG20 and RAD20 compared to S288C (p<0.001, two-tailed Student’s t-test), while the SC5314-derived progeny did not show significant changes in FoG_20_ or RAD_20_ when compared to SC5314 (p>0.05, two-tailed Student’s t-test) (**Figure 6C**). Therefore, exposure to EtBr alone does not alter FLC susceptibility; rather, the resulting mitochondrial respiratory deficiency leads to a loss of FLC tolerance and an acquisition of FLC resistance.

### Regulation of fluconazole resistance and tolerance by petites via efflux and ergosterol biosynthesis genes

We conducted investigation into the mechanisms behind the association of petite formation with a loss of FLC tolerance, characterized by a decrease in FoG20 levels, as well as an increase in FLC resistance, indicated by diminished RAD20. To delve deeper into this phenomenon, we performed a comparative analysis of the transcriptomes from one type of petite that was induced by FLC and another that was induced by EtBr, juxtaposing these profiles with those of the parental strain, S288C.

**Figure 7:**
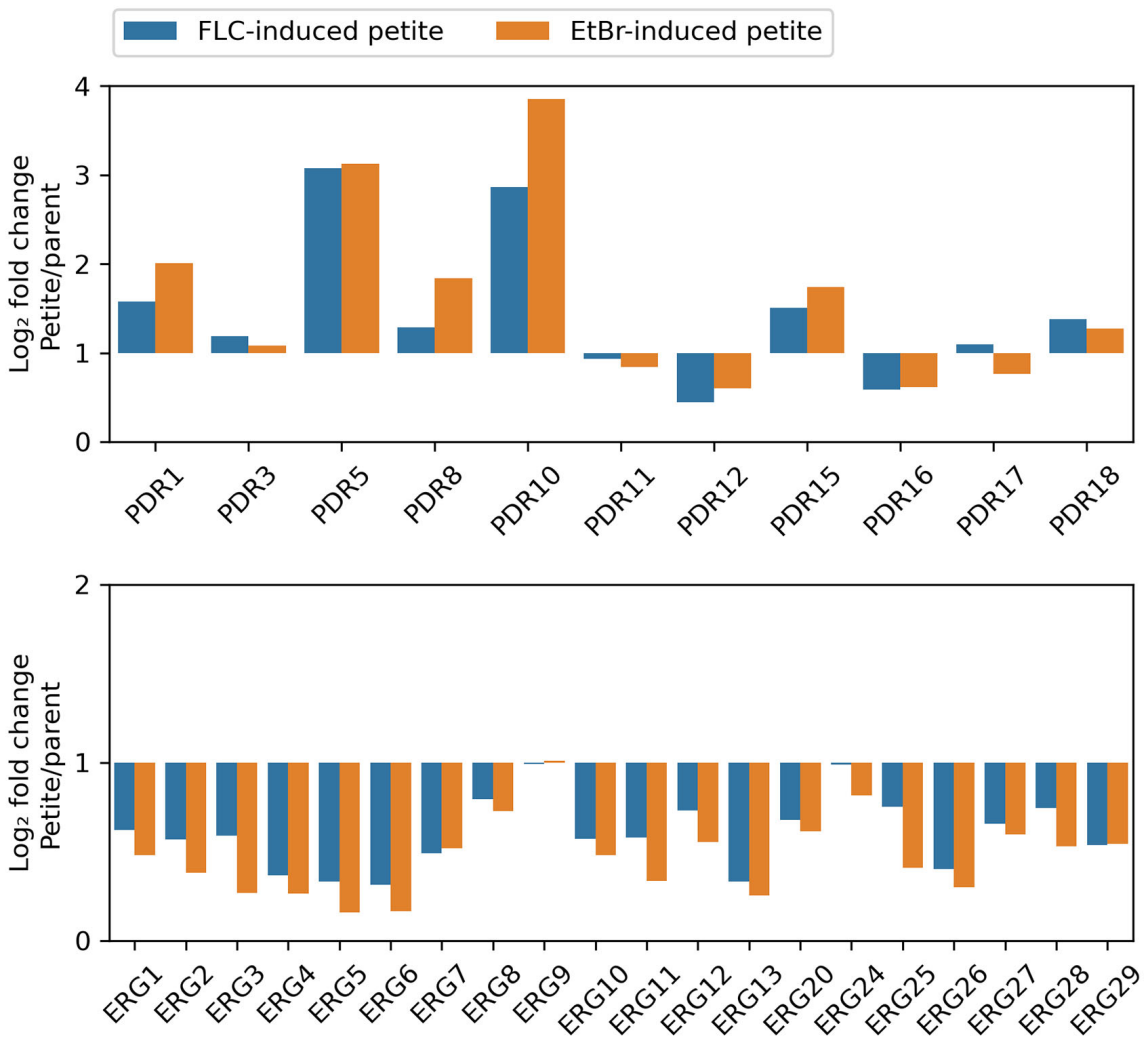
Relative Expression of Selected Genes in Petite and Wild-Type Strains The transcriptomes of two petites, derived from S288C through exposure to FLC and EtBr, respectively, were compared to that of parental strain. The figure displays the relative expressions of efflux genes (top panel) and *ERG* genes (bottom panel).

Our findings revealed a significant up-regulation of numerous efflux genes within both categories of petites throughout the genome. Notable examples of these up-regulated genes include *PDR1*, *PDR5*, *PRR8*, *PDR10*, *PDR15*, and *PDR18*, all of which are known to play critical roles in drug efflux and cellular detoxification. In stark contrast, we observed that only a small subset of efflux transporters, namely *PDR12* and *PDR16*, exhibited down-regulation. Meanwhile, *PDR3*, *PDR11*, and *PDR17* did not show any differential expression compared to the parental strain (**Figure 7**, top panel).

On the other hand, the majority of genes involved in the ergosterol biosynthetic pathway—referred to as *ERG* genes—demonstrated a pronounced down-regulation across both types of petites. This included key genes such as *ERG1*, *ERG2*, *ERG3*, *ERG4*, *ERG5*, *ERG6*, *ERG7*, *ERG8*, *ERG10*, *ERG11*, *ERG12*, *ERG13*, *ERG20*, *ERG25*, *ERG26*, *ERG27*, *ERG28*, and *ERG29*, all of which are integral to the synthesis of ergosterol. Only two *ERG* genes, *ERG9* and *ERG24*, did not exhibit any significant changes in expression levels. Importantly, none of the *ERG* genes were found to be up-regulated in either petite type, further highlighting the distinct gene expression patterns associated with petite formation (**Figure 7**, bottom panel).

**Figure 8:**
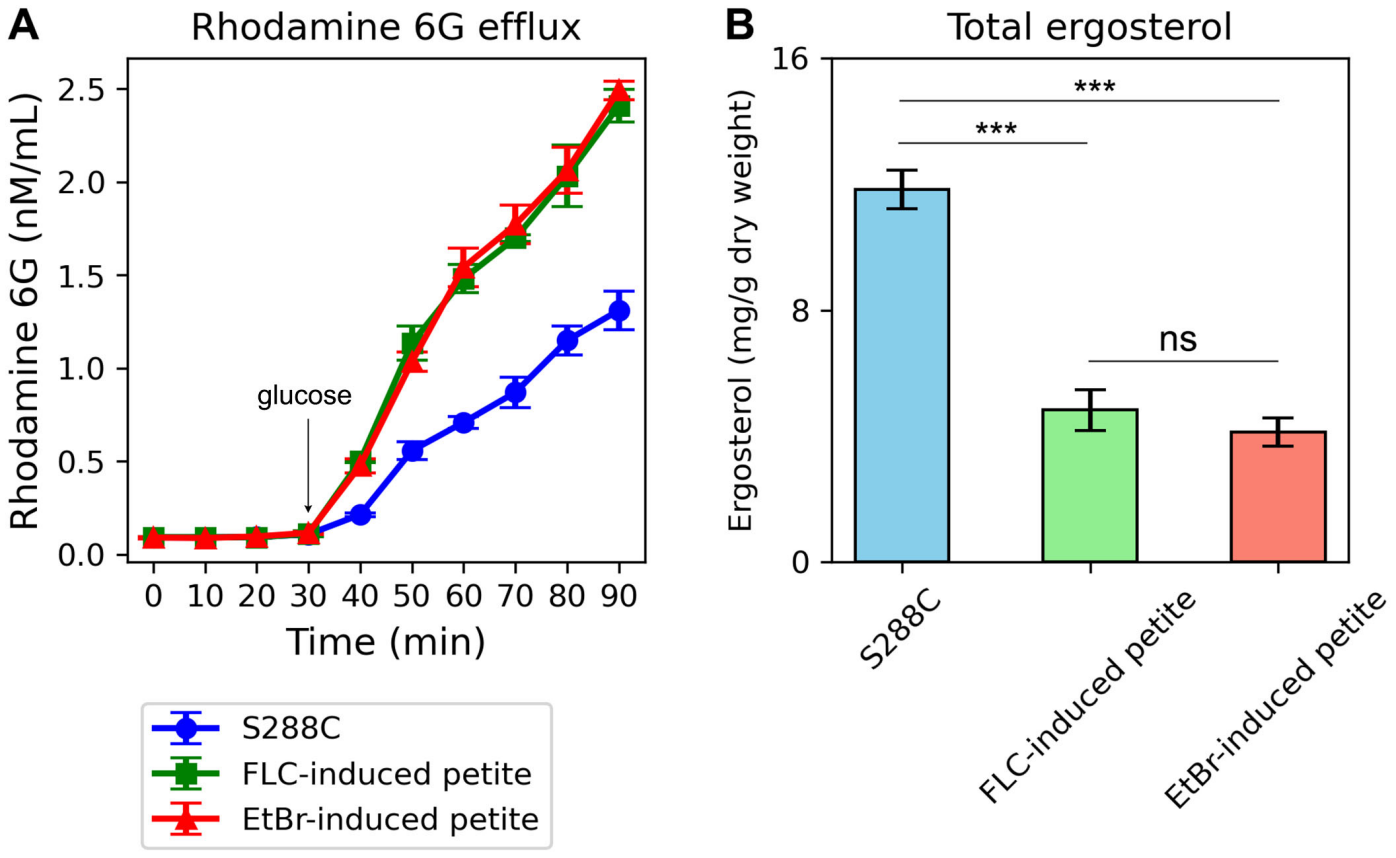
Rhodamine 6G Efflux and Total Ergosterol Content in Petite Strains Compared to the Parental Strain S288C. **(A)** The graph shows the efflux of rhodamine 6G over time in FLC-induced petites, EtBr-induced petites, and the parental strain S288C. The x-axis represents time (in minutes), and the y-axis represents the concentration of rhodamine 6G (in nM/mL) in the supernatant. Both petite strains exhibit higher rhodamine 6G efflux compared to S288C, as indicated by the increased fluorescence in the supernatant over time. Error bars represent the standard deviation of triplicate measurements. **(B)** The bar graph compares the total ergosterol content in FLC-induced petites, EtBr-induced petites, and S288C. The y-axis represents the total ergosterol content (in arbitrary units), and the x-axis labels the three strains. Both petite strains show lower ergosterol levels compared to S288C. Error bars represent the standard deviation of triplicate measurements. ***indicates p<0.001 in comparison to progenitor. ns, not significant.

To further validate these observations, we measured rhodamine 6G efflux, a key indicator of efflux pump activity. Both FLC-induced and EtBr-induced petites exhibited significantly higher rhodamine 6G efflux compared to the parental strain S288C (p < 0.001, Tukey HSD test) (**Figure 8A**). This result aligns with the up-regulation of efflux genes in petites and supports the hypothesis that enhanced efflux activity contributes to drug resistance in these strains.

Additionally, we quantified total ergosterol content in the petites and the parental strain. Consistent with the down-regulation of ERG genes, both FLC-induced and EtBr-induced petites displayed significantly lower total ergosterol levels compared to S288C (p < 0.001, two-tailed Student’s t-test) (**Figure 8B**). This reduction in ergosterol content further underscores the disruption of membrane biosynthesis in petites and its potential role in altering cellular responses to antifungal agents.

Overall, our study sheds light on the genetic underpinnings that contribute to the altered sensitivities to FLC observed in petite strains, emphasizing the crucial role of up-regulation of efflux genes and the down-regulation of ergosterol biosynthesis in this context.

## Discussion

This study presents a comparative analysis of how *S. cerevisiae* (S288C) and *C. albicans* (SC5314) adapt to FLC. Our findings indicate that FLC tolerance in both yeasts is temperature-dependent but inversely; *C. albicans* shows tolerance at 37°C and not at 30°C, while *S. cerevisiae* is tolerant at 30°C but loses this tolerance at 37°C. Unlike *C. albicans*, *S. cerevisiae* primarily develops resistance rather than tolerance to elevated FLC concentrations, with all resistant variants identified as petites. EtBr-induced petites also lost FLC tolerance and gained resistance, exhibiting up-regulation of efflux genes responsible for antifungal export and down-regulation of ergosterol biosynthesis genes. These results suggest that maintaining intact mitochondria is crucial for preserving FLC tolerance in *S. cerevisiae*.

A comparative study between *C. albicans* and *S. cerevisiae* is essential for several reasons. First, while both are yeast species, they belong to different genera and exhibit distinct biological and pathogenic characteristics; *C. albicans* is a significant human pathogen, whereas *S. cerevisiae* is commonly used as a model organism in research and baking. Studying these two species allows researchers to identify the specific mechanisms of antifungal adaptation that *C. albicans* employs in response to treatments like fluconazole, which may differ from the more limited mechanisms observed in *S. cerevisiae*. Additionally, insights gained from this comparison can shed light on the evolutionary pressures shaping pathogenic versus non-pathogenic yeasts, enhancing our understanding of fungal biology and informing the development of targeted antifungal therapies. Finally, findings from such studies can contribute to broader knowledge regarding microbial adaptation strategies, benefiting clinical practices and public health responses.

It is intriguing that temperature modulates intrinsic FLC tolerance in opposing ways for *S. cerevisiae* and *C. albicans*, given their different ecological niches and typical incubation conditions. *S. cerevisiae* is generally cultured at 30°C, where it demonstrates tolerance to FLC, while *C. albicans*, a human pathogen that inhabits the human body, typically encounters temperatures around 37°C, showing tolerance only at this higher temperature. This contrast highlights the adaptive strategies of these yeasts in response to their respective environments and underscores the importance of temperature as a factor influencing antifungal susceptibility and resistance mechanisms. Understanding these dynamics can provide valuable insights into how pathogenic yeasts adapt to host conditions and develop tolerance to antifungal treatments.

Despite the contrasting effects of temperature on FLC tolerance in *S. cerevisiae* and *C. albicans*, our study reveals that both species require calcineurin and Hsp90 for maintaining intrinsic FLC tolerance. This indicates a fundamental similarity in the molecular mechanisms underlying antifungal tolerance, despite the differences in their ecological niches and growth conditions. Calcineurin, a calcium/calmodulin-dependent phosphatase, plays a critical role in cellular signaling pathways that respond to stress, particularly in adaptation to high-temperature environments and certain antifungal agents. Its function is essential for modulating gene expression related to stress responses and maintaining cellular homeostasis under adverse conditions (Sanglard et al., 2003; Cowen, 2013). Similarly, Hsp90, a well-known chaperone protein, assists in the proper folding and stabilization of many client proteins involved in signal transduction and stress response pathways. In both yeasts, Hsp90 helps ensure that key proteins remain functional under stress, contributing to the overall resilience against antifungal treatments (Cowen and Lindquist, 2005; Cowen et al., 2006). The shared reliance on these two proteins suggests that regardless of the specific environmental pressures faced by *S. cerevisiae* and *C. albicans*, there are conserved biological pathways that facilitate intrinsic tolerance to FLC. This finding enhances our understanding of the evolutionary strategies employed by these fungi to survive antifungal challenges and highlights potential targets for therapeutic interventions. By focusing on these common pathways, we may develop more effective antifungal strategies that can mitigate tolerance in both pathogenic and non-pathogenic yeasts. Ultimately, this comparative perspective underscores the importance of studying diverse fungal species to gain insights into broader mechanisms of antifungal tolerance and adaptability.

In contrast to *S. cerevisiae*, where the formation of petites occurs relatively frequently, this phenomenon is notably rare in *C. albicans*. Although petite formation can be induced under specific conditions in *C. albicans*, it generally requires more stringent circumstances than in *S. cerevisiae*, making its occurrence uncommon (Chen and Clark-Walker, 2000; Geraghty and Kavanagh, 2003; Cheng et al., 2007a, Cheng et al., 2007b). Our study further elucidates this distinction by demonstrating that none of the FLC-tolerant adaptors derived from *C. albicans* are petites. In stark contrast, all of the FLC-resistant adaptors obtained from *S. cerevisiae* are petites. This significant finding underscores the differences between these two species not only in their capacity to form petites but also in their propensity to develop such adaptations in response to various stresses, including exposure to FLC.

The rarity of petite formation in *C. albicans* suggests a more robust mitochondrial integrity and function compared to *S. cerevisiae*, which may contribute to its survival strategies within the human host environment. This difference could indicate that *C. albicans* has evolved alternative mechanisms to cope with antifungal stress, relying on pathways other than those involving petite formation. Meanwhile, the frequent occurrence of petites in *S. cerevisiae* highlights its ability to generate metabolic diversity through mitochondrial dysfunction, enabling adaptation under stressful conditions, including drug exposure.

When petites do arise in yeast, they often confer resistance to FLC through the up-regulation of efflux genes (Henry et al., 2000; Florio et al., 2011; Gnat et al., 2021). Consistent with this, our findings revealed that both FLC- and EtBr-induced petites exhibited increased expression of multiple efflux genes, including *PDR1*, *PDR5*, *PRR8*, *PDR10*, *PDR15*, and *PDR18*. However, we also observed the downregulation of a small subset of efflux-related genes, most notably *PDR12* and *PDR16*. The downregulation of *PDR16* is particularly noteworthy, as its deletion has been previously demonstrated to increase azole susceptibility in *S. cerevisiae*. *PDR16* is involved in lipid homeostasis and membrane composition, which are critical for the proper localization and function of efflux pumps such as *PDR5* (Holic et al., 2014). In the context of petites, the downregulation of *PDR16* may reflect a broader disruption of lipid metabolism and membrane dynamics associated with mitochondrial dysfunction. While *PDR16* downregulation would typically be expected to increase azole susceptibility, the concurrent upregulation of other efflux transporters (e.g., *PDR5*, *PDR10*, and *PDR15*) in petites may compensate for this effect, leading to the observed increase in FLC resistance. This suggests a complex interplay between lipid metabolism, efflux pump activity, and drug resistance in petites.

Typically, exposure to FLC promotes the expression of ergosterol biosynthesis genes (*ERG* genes) in fungi (Henry et al., 2000; Florio et al., 2011; Gnat et al., 2021). However, our study indicates that petites display reduced expression of most *ERG* genes, including *ERG11*, which encodes the target protein of FLC. We propose that sufficient levels of ergosterol are essential for maintaining FLC tolerance, suggesting a critical link between ergosterol biosynthesis and antifungal susceptibility in *S. cerevisiae*. This highlights the unique mechanisms of adaptation in these yeasts and the potential implications for antifungal treatment strategies.

## Conclusion

In conclusion, our results underscore the opposite impact of temperature on FLC tolerance in *S. cerevisiae* and *C. albicans*; while elevated temperatures adversely affected *S. cerevisiae*, they appeared to enhance FLC tolerance in *C. albicans*. This study highlights the distinct evolutionary responses to FLC in *S. cerevisiae* compared to previous findings in *C. albicans*. We observed that rapid petite formation in *S. cerevisiae* resulted in the up-regulation of efflux genes and the down-regulation of *ERG* genes, which likely contributed to a gain in FLC resistance while simultaneously leading to a loss of FLC tolerance. Given the shifting epidemiology of candidemia to include an increasing number of NAC species, it is crucial to further characterize FLC tolerance within these NAC species. Understanding these dynamics will be vital for developing effective treatment strategies and addressing the challenges posed by antifungal resistance in diverse fungal pathogens.

## Data Availability

The data presented in the study are deposited in the ArrayExpress repository, accession number E-MTAB-14553.
